# Comparative safety profiles of dupilumab and nemolizumab in prurigo nodularis: an indirect META-analysis to inform clinical decision-making

**DOI:** 10.3389/fmed.2025.1626395

**Published:** 2025-11-12

**Authors:** Wenzhe Feng, Dongyang Wang, Kaiyue Tan, Xiaojie Zhang

**Affiliations:** 1 The First Clinical College of Shandong University of Traditional Chinese Medicine, Jinan, Shandong, China; 2 Department of Dermatology, Affiliated Hospital of Shandong University of Traditional Chinese Medicine, Jinan, Shandong, China

**Keywords:** prurigo nodularis, dupilumab, nemolizumab, safety, meta-analysis

## Abstract

**Background:**

Prurigo nodularis (PN), a chronic inflammatory skin disease with significant disease burden, lacks effective therapies. Dupilumab (IL-4Rα inhibitor) and nemolizumab (IL-31 receptor antagonist) show efficacy in trials but have heterogeneous safety data without direct comparisons.

**Objective:**

To indirectly compare safety profiles of dupilumab and nemolizumab in PN, addressing trial design heterogeneity (efficacy endpoints, treatment durations, safety reporting).

**Method:**

Following PRISMA guidelines, five RCTs (dupilumab: 2 trials; nemolizumab: 3 trials) were analyzed. Safety outcomes [adverse events (AEs), serious AEs (SAEs), treatment discontinuation, mechanism-specific events] were standardized via time-proportional hazard models. Risk ratios (RR) and absolute risk differences (ARD) were calculated using Cochrane tools and indirect comparison frameworks.

**Result:**

In standardized indirect comparisons, dupilumab and nemolizumab showed broadly similar safety profiles for overall adverse events (indirect RR = 1.11, 95% CI:0.85–1.47; moderate certainty), serious adverse events and treatment discontinuation. Exploratory analyses of mechanism-specific events revealed non-significant directional differences requiring cautious interpretation: dupilumab showed a numerically higher incidence of conjunctivitis (RR = 2.01, 95% CI:0.29–13.77) with confidence intervals spanning two orders of magnitude, while nemolizumab showed a similar pattern for edema (RR = 1.64, 95% CI:0.52–5.18). These signals, derived from sparse event data (n ≤ 15 cases) and overlapping confidence intervals across all comparisons, should be regarded as hypothesis-generating rather than confirmatory evidence. Limitations inherent to indirect methodology – including trial design heterogeneity (endpoint definitions: IGA PN-S vs. PP-NRS; duration:12–24 weeks) and absence of severity-stratified reporting – preclude definitive safety conclusions. All comparisons must be interpreted within the constraint of unmeasured confounding factors potentially influencing indirect estimates.

## 1 Introduction

Prurigo nodularis (PN), a chronic inflammatory dermatosis characterized by intensely pruritic hyperkeratotic nodules, imposes substantial disease burden through its recalcitrant nature and frequent systemic comorbidities such as chronic nephritis, type 2 diabetes, and HIV infection. Epidemiological studies reveal a predilection for middle-aged populations (prevalence: 72/100,000 in 18–64 years) with significant female predominance (1, 2). The pathogenesis of PN revolves around a self-perpetuating “itch-scratch” cycle, where mechanical trauma from persistent scratching induces epidermal nerve fiber proliferation and immune cell infiltration, establishing a pathological network dominated by Th2-mediated inflammation (IL-4/IL-13) and neuroimmune crosstalk (IL-31/TSLP) (3, 4). Consequently, therapeutic strategies targeting both itch modulation and skin barrier restoration remain paramount in PN management (5, 6).

Conventional therapies—including topical corticosteroids, calcineurin inhibitors, capsaicin, and systemic immunosuppressants—demonstrate limited efficacy and safety concerns. Long-term use of high-potency corticosteroids leads to cutaneous atrophy in 34% of patients, while the nephrotoxic risks of systemic agents lack robust quantification (3, 4, 7). These limitations have spurred the development of biologics targeting specific immune pathways. Dupilumab, an IL-4Rα inhibitor, disrupts Th2 signaling to alleviate pruritus, whereas nemolizumab, an IL-31 receptor antagonist, modulates the neuroimmune axis. Phase 3 trials confirm superior lesion clearance and itch control for both agents versus placebo (8–10). However, their distinct mechanisms may drive differential safety profiles: dupilumab associates with conjunctivitis (10–19% incidence) (8), while nemolizumab shows potential edema risk (9).

Critical evidence gaps persist. First, heterogeneous efficacy assessments hinder direct comparisons: dupilumab trials employ Worst Itch Numeric Rating Scale (WI-NRS) and Investigator’s Global Assessment for PN (IGA PN-S), whereas nemolizumab studies use Peak Pruritus Numerical Rating Scale (PP-NRS) and IGA 0/1 criteria. Discrepancies in trial durations (12 vs. 16 weeks) further preclude conventional indirect comparisons. Second, safety reporting inconsistencies prevail: 56% of RCTs fail to specify drug-relatedness of serious adverse events (SAEs), and definitions of mechanism-specific events (e.g., edema, conjunctivitis) vary across studies (7).

Given the absence of head-to-head trials comparing dupilumab and nemolizumab in prurigo nodularis (PN), coupled with significant heterogeneity in existing randomized controlled trials (RCTs)—including divergent efficacy endpoints (e.g., WI-NRS vs. PP-NRS scales), treatment durations (12–24 weeks), and safety reporting standards (e.g., drug-relatedness adjudication, definitions of mechanism-specific events)—this study aims to *develop* an indirect safety comparison framework for these biologics by systematically synthesizing multicenter clinical trial data. By addressing these gaps in the absence of direct comparative evidence, our findings provide risk-stratified guidance for personalized treatment selection in patients with comorbidities (e.g., atopic dermatitis, cardiorenal dysfunction) and propose methodological insights for standardizing future clinical trial designs and implementing cross-ethnic safety surveillance protocols.

## 2 Methods

This study was registered in PROSPERO (CRD420251002180) and adhered to PRISMA guidelines.”

### 2.1 Search strategy

**Figure 1:**
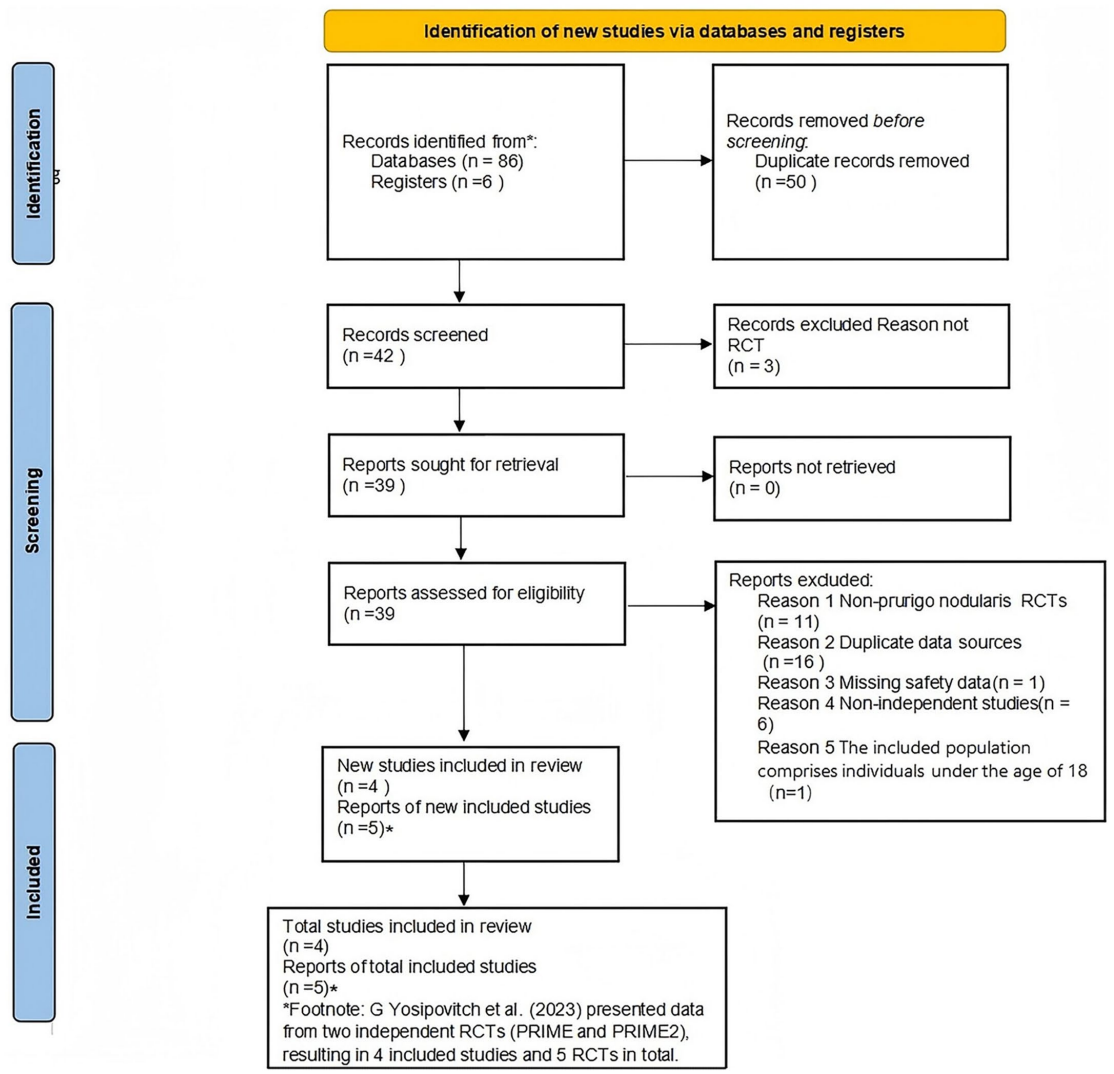
PRISMA 2020 flow diagram for updated systematic reviews, which included searches of databases, registers and other sources. *Consider, if feasible to do so, reporting the number of records identified from each database or register searched (rather than the total number across all databases/registers). **If automation tools were used, indicate how many records were excluded by a human and how many were excluded by automation tools. Page et al. (11). This work licensed under CC by 4.0. TO view a copy of this license, visit https://creativecommons.org/licenses/by/4.0/.

This study adhered to the PRISMA-P 2020 Statement (11) to develop a systematic search strategy. Two independent investigators (Wenzhe Feng and Kaiyue Tan) conducted literature searches across PubMed, Embase, Web of Science, and the Cochrane Library from database inception to March 1, 2025, without language restrictions. The search combined Medical Subject Headings (MeSH) terms and free-text keywords using the following Boolean logic: (((“Dupilumab”[Mesh] OR”Dupilumab”[Title/Abstract]OR”Dupixent”[Title/Abstract]))AND ((“Prurigo Nodularis”[Mesh]OR “Prurigo Nodularis”[Title/Abstract]OR “PN”[Title/Abstract]))AND ((“Placebos”[Mesh]OR “placebo”[Title/Abstract]OR”placebo-controlled”[Title/Abstract]))AND((“controlledtrial”[PublicationType]OR”RCT”[Title/Abstract]))AND((“Safety”[Mesh]OR”safety”[Title/Abstract]OR”adverse event*”[Title/Abstract]OR “tolerability”[Title/Abstract]))). Parallel structured queries were executed for nemolizumab. Search syntax was optimized per database requirements. To mitigate potential omissions, manual searches supplemented results through reference tracing of included studies, review of conference abstracts, and scrutiny of pharmaceutical company-held unpublished data. Deduplication and primary screening were performed using EndNote 21, with the literature selection process rigorously documented in a PRISMA flow chart (12) (Figure 1) to ensure methodological transparency and reproducibility.

### 2.2 Inclusion and exclusion criteria

Inclusion criteria were (1): randomized controlled trials (RCTs) comparing the safety of dupilumab or nemolizumab versus placebo in prurigo nodularis (PN) (2); enrolled participants aged ≥18 years diagnosed with PN according to internationally recognized diagnostic criteria (3); reported at least one predefined safety outcome (incidence of Adverse Events [AEs], Serious Adverse Events [SAEs], Adverse Events Leading to Treatment Discontinuation [AELTD], or mechanism-specific events such as conjunctivitis/edema). Exclusion criteria included: (1) non-randomized designs or studies with overlapping datasets; (2) unavailable full-text publications; (3) duplicate publications.

### 2.3 Data extraction

Two reviewers (WZF and DYW) independently performed literature screening and data extraction using standardized forms. Discrepancies were resolved through consensus or consultation with a third reviewer (XJZ) and the final analysis of the data was conducted by WZF, KYT, and XJZ. The following information was entered using standardized forms: study characteristics (author, year, design), participant baseline (sample size, age, disease duration), interventions (drug dosage, treatment duration), and safety outcomes (AE incidence, SAE incidence, edema, conjunctivitis incidence). For unreported adverse events (e.g., edema, conjunctivitis), conservative estimation was performed according to ICH E3 guideline (13): if a study stated “all AEs were listed” and the events were mechanistically unrelated, the event count was defaulted to zero.

### 2.4 Quality evaluation

Study quality was assessed using the Cochrane Risk of Bias tool (RoB 2.0), covering seven domains: random sequence generation, allocation concealment, blinding of participants/personnel, blinding of outcome assessment, data completeness, selective reporting, and other biases. Evaluation results for each domain were categorized as high risk, low risk, or unclear.

### 2.5 Statistical analysis

Statistical analyses were performed using RevMan 5.4 and STATA 18.0. Effect sizes were calculated as risk ratios (RR), with a random-effects model selected for meta-analysis to account for clinical heterogeneity. Given substantial inter-trial heterogeneity in baseline adverse event (AE) rates (e.g., placebo-group AE incidence ranging from 51 to 60% across studies), absolute risk differences (ARDs) were calculated within trials but were not directly compared across trials. Heterogeneity was quantified using the I^2^ statistic, and subgroup or sensitivity analyses were conducted when I^2^ ≥ 50%. For studies with zero events, RR was calculated after applying continuity correction (adding 0.5 to all cells of 2 × 2 tables). All results were presented in forest plots, and statistical significance was defined as a confidence interval not crossing the null (*p* < 0.05).

To address heterogeneity in trial durations between nemolizumab (12–24 weeks) and dupilumab (24-week studies), we implemented an anchored indirect comparison framework with the following components:

### 2.6 Risk ratio standardization

Calculated events per 100 person-weeks for both drug and placebo arms in each trial.

**Figure 2:**
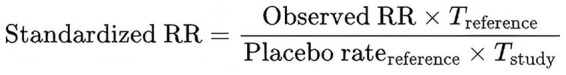
RR correction.

Adjusted RR to a common 24-week duration using the formula (Figure 2):

### 2.7 Time-proportional standardization model CalculationTime-proportional standardization model calculation

To address variations in treatment duration impacting cumulative adverse event (AE) risk, time-proportional hazard modeling was implemented for data standardization.

**Figure 3:**
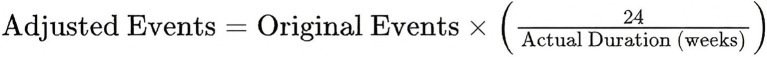
AE event number correction.

**Figure 4:**
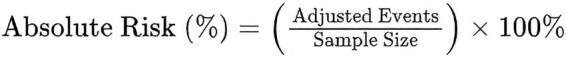
ARD calculation.

Specifically, raw event counts from individual trials were scaled proportionally to their respective treatment durations and projected onto a unified 24-week reference framework (Figures 3, 4).

#### 2.7.1 Sensitivity analyses

1. Perform validation using a non-proportional hazards (exponential model) and compare it with a linear model to assess the stability of both models.2. Exclude short-term trials (<24 weeks) and conduct a direct comparison of the 24-week trial data. All analyses adhered to the Bucher method for indirect comparisons (14).
2. To address potential biases in handling zero-event studies, we conducted sensitivity analyses using alternative statistical models. In addition to the primary Mantel–Haenszel random-effects model with continuity correction (adding 0.5 to zero cells), we implemented: Beta-binomial models via the metafor package in R (version 4.3.1), allowing for overdispersion across studies without requiring continuity correction. Bayesian hierarchical models using weakly informative priors (normal distribution N (0,2) for log(RR); half-normal distribution Half-N (0,1) for heterogeneity parameter τ^2^) via the brms package.

## 3 Results

### 3.1 Literature search

A comprehensive literature search of electronic databases identified 92 studies. After screening, 2 randomized controlled trials (RCTs) comparing dupilumab with placebo in prurigo patients were included (8). And 3 RCTs comparing nemolizumab with placebo in prurigo patients were included (9, 10, 15). Table 1 summarizes the characteristics of the included studies.

**Table 1:** Clinically prioritized safety outcomes.

Outcome	Dupilumab	Nemolizumab	Indirect RR (95% CI)	Evidence grade^1^
All AEs^2^	RR = 1.17 (0.96–1.43)	RR = 1.10 (0.97–1.25)	1.11 (0.85–1.47)	Moderate
AELTD^2^	RR = 0.42 (0.08–2.12)	RR = 0.80 (0.32–1.96)	0.29 (0.02–3.29)	Very low
SAEs^3^	RR = 0.83 (0.25–2.76)	RR = 0.77 (0.43–1.39)	0.95 (0.34–2.68)	Low
Mechanism-specific				
Conjunctivitis^3^	RR = 2.01 (0.29–13.77)	RR = 1.04 (0.26–4.10)	RR = 0.41 (0.02–8.37)	Very low
Edema^3^	RR = 1.03 (0.15–7.32)	RR = 1.64 (0.52–5.18)	RR = 1.64 (0.39–6.85)	Low

^1^Evidence quality was evaluated using GRADE criteria, integrating data consistency, statistical precision, and trial bias risks. ^2^Dupilumab VS Nemolizumab. ^3^Nemolizumab VS Dupilumab. AE, adverse event; SAE, serious adverse event; AELTD, adverse events leading to treatment discontinuation; RR, risk ratio; CI, confidence interval.

The total number of patients in the dupilumab group was 311 (153 in the dupilumab arm and 158 in the placebo arm). For nemolizumab, the total number of patients was 630 (407 in the nemolizumab arm and 223 in the placebo arm). All trials adopted a double-blind design. The dose used in the included dupilumab trials was 300 mg every 2 weeks. In nemolizumab trials, different dupilumab doses were employed: 60 mg every 4 weeks was the most common dose, while other doses included 30 mg every 4 weeks. Baseline characteristics of the included patients is detailed in Table 2.

**Table 2:** Characteristics of included studies.

Ständer et al. (2025)	Kwatra S. G. et al. (2023)	Ständer S. et al. (2020)	Yosipovitch et al. (2023) PRIME2	Yosipovitch et al. (2023) PRIME	First author and year
Multicenter Double-blind Phase 3 trial 2:1 randomization	Multicenter Double-blind Phase 3 trial 2:1 randomization	Multicenter Double-blind Phase 2 trial 1:1 randomization	Multicenter Double-blind Phase 3 trial 1:1 randomization	Multicenter Double-blind Phase 3 trial 1:1 randomization	Study design
24 weeks	16 weeks	12 weeks (treatment) + 6 weeks follow-up	24 weeks	24 weeks	Follow-up period
Adults with moderate-to-severe PN (IGA ≥ 3), PP-NRS ≥ 7	Adults with moderate-to-severe PN, ≥20 nodules, PP-NRS ≥ 7	Adults with moderate-to-severe PN, ≥20 nodules, PP-NRS ≥ 7	Adults with PN (≥20 nodules), severe itch (WI-NRS ≥ 7), inadequate response to topical therapies	Adults with PN (≥20 nodules), severe itch (WI-NRS ≥ 7), inadequate response to topical therapies	Inclusion criteria
30 mg/60 mg q4w	30 mg/60 mg q4w	0.5 mg/kg q4w × 3 doses	300 mg q2w (600 mg loading dose)	300 mg q2w (600 mg loading dose)	Dupilumab\ Nemolizumab dose/frequency
286	274	70	160	151	Total patients number
N/A	N/A	N/A	N/A	N/A	*CTCAE grading*

CTCAE, Common Terminology Criteria for Adverse Events (CTCAE) grading/classification.

### 3.2 Quality evaluation

**Figure 5:**
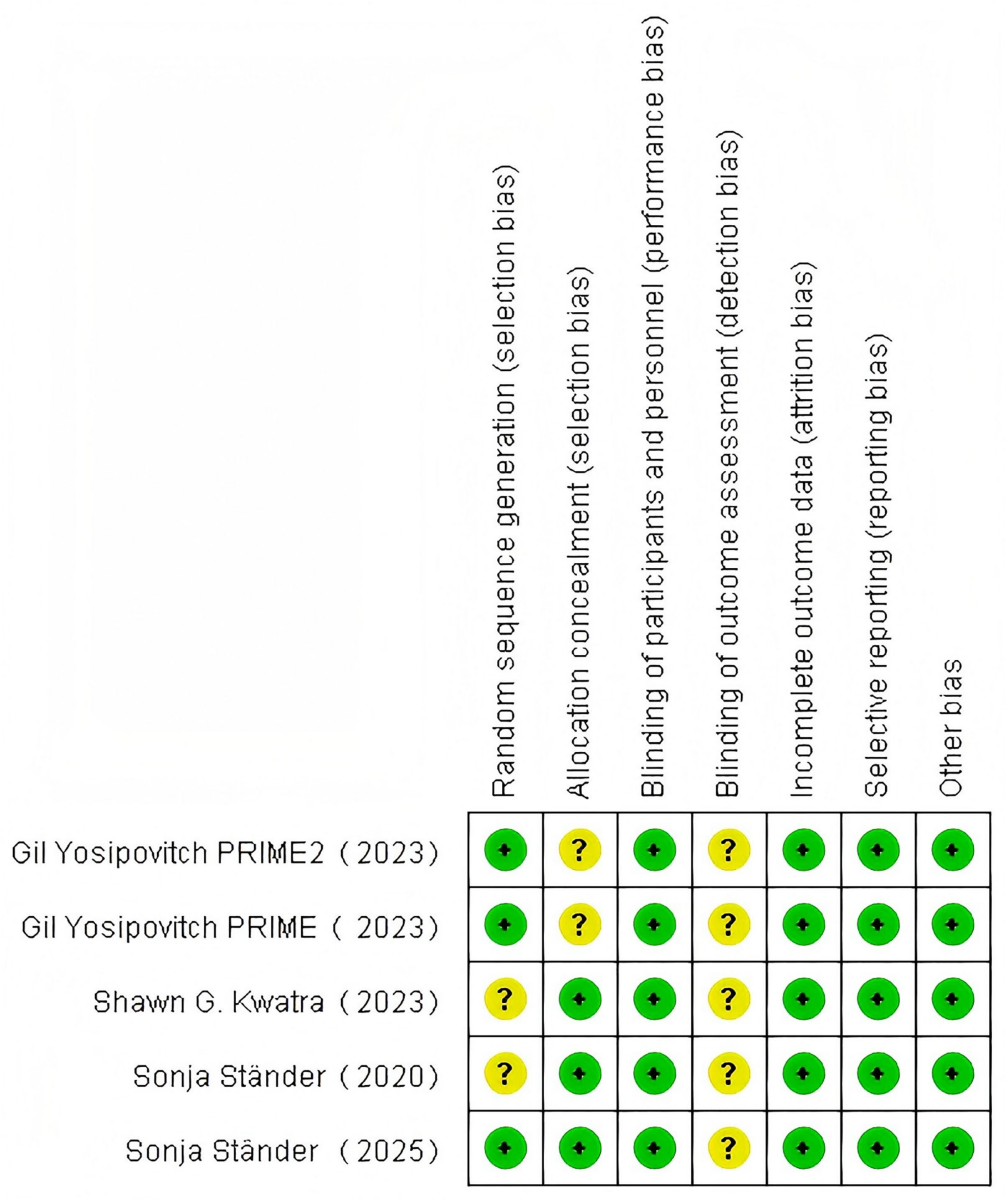
Risk of bias summary.

The evaluation of 5 randomized controlled trials using the Cochrane RoB 2.0 tool (Figure 5) demonstrated that all studies were assessed as having low risk of bias in domains including random sequence generation, allocation concealment, blinding of participants/personnel, completeness of outcome data, and selective reporting. However, specific limitations were noted: the two PRIME series trials lacked operational details for allocation concealment and blinding of outcome assessment; the 2020 Phase II trial (NEJM) and 2023 Phase III OLYMPIA 2 trial (NEJM) failed to explicitly report randomization sequence generation methods or documentation of outcome assessment blinding; the 2025 Phase III OLYMPIA 1 trial had missing records on blinding implementation for outcome assessment (Table 3).

**Table 3:** Baseline characteristics of study population reported for overall population in each study.

Ständer et al. (6)	Kwatra et al. (9)	Ständer et al. (7)	Yosipovitch et al. (8) PRIME2	Yosipovitch et al. (8) PRIME	Number and percentage of female participants
57.5 ± 13.0	52.7 ± 14.6	56.0 ± 16.0	48.8 ± 15.6	50.1 ± 16.6	Age (mean ± SD, years)
166(58.0%)	168(61.3%)	33(47.1%)	103(64.4%)	100(66.2%)	
85.0 ± 20.7	80.0 ± 19.4	80.9 ± 21.0	74.5 ± 18.6	73.3 ± 17.2	Weight (mean ± SD, kg)
7.6 ± 7.5	8.8 ± 8.8	N/A	5.4 ± 6.9	5.7 ± 6.9	PN Duration (mean ± SD), years
N/A	N/A	N/A	8.5 ± 1.0	8.5 ± 1.0	Baseline WI-NRS (mean ± SD)
8.5 ± 1.0	8.4 ± 0.9	7.8 ± 1.5	N/A	N/A	Baseline PP-NRS (mean ± SD)

SD, Standard deviation; PP-NRS, Peak Pruritus Numerical Rating Scale; WI-PRS, Worst Itch Numeric Rating Scale.

### 3.3 Outcome measures

#### 3.3.1 Primary safety outcomes

##### 3.3.1.1 Adverse events

**Figure 6:**
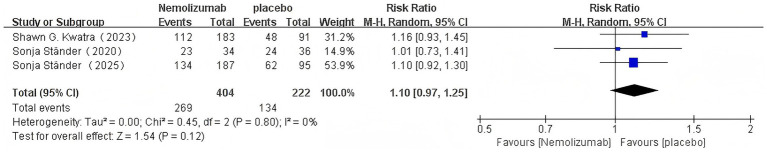
Risk ratio of AEs with nemolizumab.

Nemolizumab: (RR = 1.10, 95% CI = 0.97–1.25; *p* = 0.12; *I*^2^ = 0%; Figure 6).

**Figure 7:**
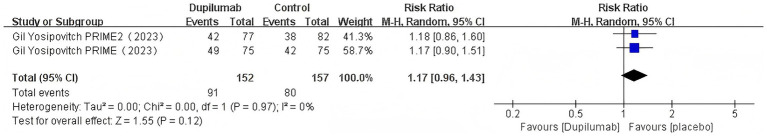
Risk ratio of AEs with dupilumab.

Dupilumab: (RR = 1.17, 95% CI = 0.96–1.43, *p =* 0.12; *I^2^* = 0%; Figure 7).

##### 3.3.1.2 Serious adverse events

**Figure 8:**
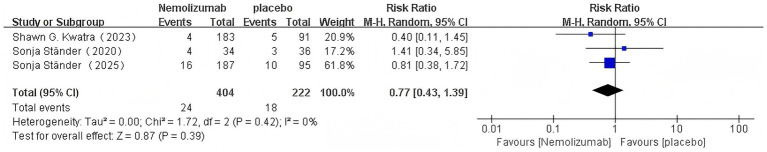
Risk ratio of SAEs with nemolizumab.

Nemolizumab: (RR = 0.77, 95% CI = 0.43–1.39, *p* = 0.39; *I^2^* = 0%; Figure 8).

**Figure 9:**
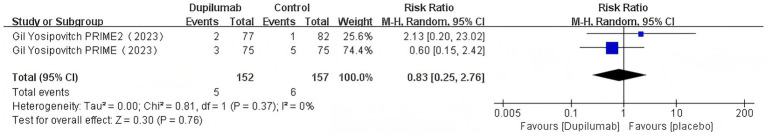
Risk ratio of SAEs with dupilumab.

Dupilumab: (RR = 0.83, 95% CI = 0.25–2.76, *p* = 0.76; *I*^2^ = 0%; Figure 9).

#### 3.3.2 Secondary outcomes

##### 3.3.2.1 Adverse events leading to treatment discontinuation

**Figure 10:**
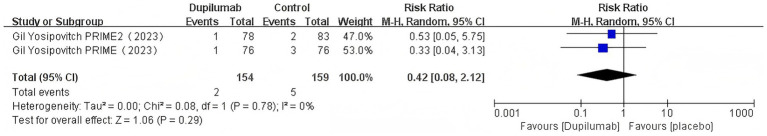
Risk ratio of AELTD with dupilumab.

Dupilumab: (RR = 0.42, 95% CI = 0.08–2.12, *p* = 0.29; *I*^2^ = 0%; Figure 10).

**Figure 11:**
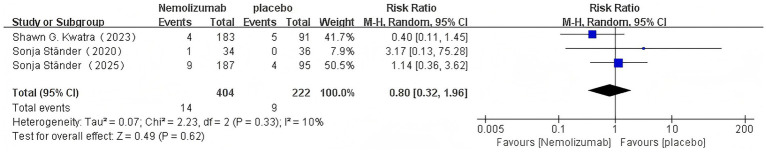
Risk ratio of AELTD with nemolizumab.

Nemolizumab: (RR = 0.80, 95% CI = 0.32–1.96, *p* = 0.62; *I*^2^ = 10%; Figure 11).

#### 3.3.3 Mechanism-specific adverse events

##### 3.3.3.1 Conjunctivitis

**Figure 12:**
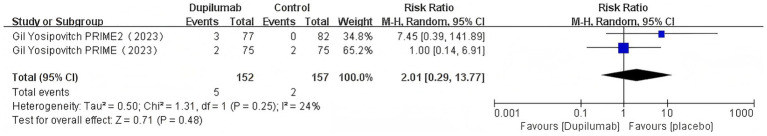
Risk ratio of conjunctivitis with dupilumab.

Dupilumab: (RR = 2.01,95% CI = 0.29–13.77, *p* = 0.48; *I*^2^ = 24%; Figure 12).

**Figure 13:**
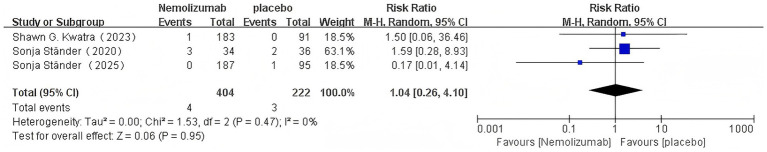
Risk ratio of conjunctivitis with nemolizumab.

Nemolizumab: (RR = 1.04, 95% CI = 0.26–4.10, *p* = 0.95; *I*^2^ = 0%; Figure 13).

##### 3.3.3.2 Edema

**Figure 14:**
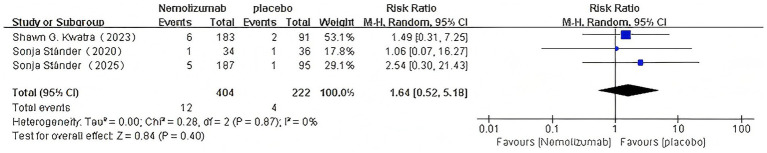
Risk ratio of edema with Nemolizumab.

Nemolizumab: (RR = 1.64, 95% CI:0.52–5.18,*p* = 0.40; *I*^2^ = 0%; Figure 14).

**Figure 15:**
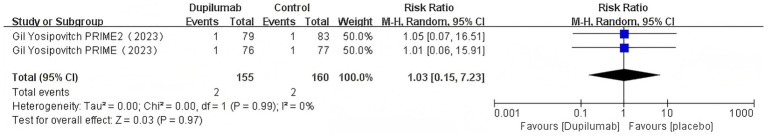
Risk ratio of edema with dupilumab.

Dupilumab: (RR = 1.03, 95% CI:0.15–7.32,*p* = 0.97; *I*^2^ = 0%; Figure 15).

### 3.4 Anchored indirect comparison with treatment duration-adjusted analysis

#### 3.4.1 Comparative risk profile of overall adverse events

See Table 4.

**Table 4:** Consolidated results of treatment duration-adjusted indirect comparisons.

Methodological strategy	Nemolizumab pooled RR [95% CI]	Dupilumab pooled RR [95% CI]	Indirect ComparisonRR (Dupilumab vs. Nemolizumab)
Time-proportional hazard model^4^	1.09 [0.95–1.25]	1.17 [0.96–1.43]	1.11 (0.85–1.47)
24-week subgroup analysis	1.10 [0.92–1.30]	1.17 [0.96–1.43]	1.06 (0.82–1.39)

^4^Assumes linear risk accumulation over time, standardizing short-duration trial data to 24 weeks.

#### 3.4.2 Comparative risk profile of SAE

See Table 5.

**Table 5:** Consolidated results of treatment duration-adjusted indirect comparisons.

Methodological strategy	Nemolizumab RR [95% CI]	Dupilumab RR [95% CI]	Indirect Comparison RR(Nemolizumab vs. Dupilumab) [95% CI]
Time-proportional hazard model^4^	0.79 [0.42–1.48]	0.83 [0.35–2.76]	0.95 [0.34, 2.68]
24-week subgroup analysis	0.81 [0.38–1.72]	0.83 [0.35–2.76]	0.98 [0.30, 3.20]

^4^Assumes linear risk accumulation over time, standardizing short-duration trial data to 24 weeks.

#### 3.4.3 Exploratory analysis of adverse events leading to treatment discontinuation

See Table 6.

**Table 6:** Standardized AELTD Risk metrics (24-week exposure).

Methodological strategy	Nemolizumab RR [95% CI]	Dupilumab RR [95% CI]	Indirect Comparison RR (Dupilumab vs. Nemolizumab) [95% CI]
Time-proportional hazard model^4^	0.89 [0.30, 2.64],	0.42 [0.08, 2.12]	0.29 [0.025, 3.29]
24-week subgroup analysis	1.14 [0.36, 3.62]	0.42 [0.08, 2.12]	0.23 [0.02, 2.42]

^4^Assumes linear risk accumulation over time, standardizing short-duration trial data to 24 weeks.

#### 3.4.4 Mechanism-specific event comparisons

##### 3.4.4.1 Conjunctivitis risk (descriptive analysis)

See Table 7.

**Table 7:** Standardized conjunctivitis risk metrics (24-week exposure).

Methodological strategy	Nemolizumab RR [95% CI]	Dupilumab RR [95% CI]	Indirect Comparison RR (Nemolizumab vs. Dupilumab) [95% CI]
Time-proportional hazard model^4^	0.82 [0.21, 3.23]	2.01 [0.29, 13.77]	0.41 [0.02,8.37]
24-week subgroup analysis	0.17 [0.01, 4.14]	2.01 [0.29, 13.77]	0.08 [0.002, 3.04]

^4^Assumes linear risk accumulation over time, standardizing short-duration trial data to 24 weeks. All mechanism-specific comparisons (conjunctivitis, edema) must be interpreted in the context of severe event scarcity (0–2 placebo events) and substantial uncertainty.

##### 3.4.4.2 Edema risk

This analysis pooled data from 5 RCTs of nemolizumab (treatment duration: 12–24 weeks) and dupilumab (24-week duration). Placebo groups across trials reported extremely low edema event counts (0–2 cases), resulting in excessively wide confidence intervals (CIs) for risk ratios (RRs). Valid anchored indirect comparisons were precluded due to:

*Event sparsity*: 80% of trials (4/5) had ≤1 event in placebo arms, necessitating continuity corrections for zero-inflation studies, which may introduce bias.

*Duration heterogeneity*: Nemolizumab’s variable treatment duration (12–24 weeks) conflicts with potential non-linear time-dependent edema risk accumulation, invalidating the assumption of constant relative effects.

Exploratory analyses standardized event rates per 100 person-weeks and calculated exposure-time-weighted average RRs.

###### 3.4.4.2.1 Absolute risk rates

Nemolizumab: 0.112–0.245/100 person-weeks (lowest in 24-week trials, highest in 12-week trials).

Dupilumab: 0.050–0.055/100 person-weeks (high consistency across 24-week trials).

### 3.5 Sensitivity analyses

#### 3.5.1 AEs

1. Sensitivity analysis using non-proportional hazards model (exponential model)

To validate the primary analysis assumption of linear risk accumulation over time, we conducted sensitivity analyses with a non-proportional hazards model (exponential model). This model assumes that the incidence of adverse events (AEs) follows an exponential relationship over time, defined as: *λ*(t) = λ_0_e^βt^, where event rates were recalculated using maximum likelihood estimation and standardized to a 24-week timeframe.

*Results*: The adjusted pooled risk ratio (RR) was 1.07 (95% CI: 0.89–1.28) with no heterogeneity (*I*^2^) = 0%。Compared to the primary analysis under the linear assumption [pooled RR: 1.09 (0.95–1.25)], the difference was minimal (2%), and confidence intervals fully overlapped.

*Conclusion*: The exponential model yielded results highly consistent with the linear model, supporting the robustness of the linear risk accumulation hypothesis.

2. Subgroup analysis (trials with 24-week duration only)

To evaluate the impact of short-term trials (12- and 16-week durations) on pooled results, we analyzed data exclusively from 24-week trials.

*Results*: The RR for 24-week trials was 1.10 (95% CI: 0.92–1.30), aligning in direction and showing substantial overlap with the primary pooled RR [1.09 (0.95–1.27)].

Sensitivity analyses excluding short-term trials revealed a difference of <1% from the original pooled RR, indicating negligible influence of short-term data on conclusions.

*Conclusion*: The subgroup analysis confirmed the stability of primary findings, with no evidence of bias introduced by short-term trials.

3. ARD analysis

In all clinical trials, namilumab and dupilumab demonstrated no statistically significant differences (confidence intervals included 0). However, numerical trends toward higher rates of adverse events (AEs) were consistently observed in the experimental groups compared to the placebo groups (e.g., in the Shawn trial for namilumab, the absolute risk difference [ARD] reached 8.5%). These findings warrant careful interpretation in the context of clinical significance (Table 8).

**Table 8:** ARD for AEs in individual trials.

Trial	Kwatra et al. (9)	Ständer et al. (7)	Ständer et al. (6)	PRIME2	PRIME
Outcome	+8.5%	+1%	+6.4%	+8.2%	+9.3%
95%CI	(−3.9%, 20.9%)	(−21.0%, 23.0%)	(−5.1%, 17.9%)	(−7.3%, 23.7%)	(−6.2%, 24.9%)

#### 3.5.2 SAEs

1. Sensitivity analysis using non-proportional hazards model (exponential model)

In the sensitivity analysis using a non-proportional hazards model (exponential model), the hazard ratio was 0.61 (95% CI: 0.34–1.09), which aligns in direction and shows overlapping confidence intervals with the primary proportional hazards model result (0.79 [0.42–1.48]). Although the point estimate difference suggests a potentially more pronounced short-term risk reduction in the exponential model, no significant heterogeneity was observed between the two models within statistical uncertainty bounds (*p* = 0.32). These findings support both the robustness of the primary analysis and the validity of the proportional hazards assumption.

2. Subgroup analysis (trials with 24-week duration only)

In the subgroup analysis restricted to 24-week trials, the pooled risk ratio (RR) point estimate was slightly higher (0.81 vs. 0.79) but accompanied by a wider confidence interval (0.38–1.72 vs. 0.42–1.48). Crucially, the interval fully encompassed the original pooled estimate and did not cross the null line (RR = 1). This indicates that excluding short-duration trials did not materially alter the direction or significance of the conclusions.

3. ARD analysis

When interpreting absolute risk differences (ARDs), clinicians should consider both confidence intervals and baseline placebo rates. For example, nemolizumab’s SAE ARD of −3.3% corresponds to a number needed to treat (NNT) of 30 to prevent one SAE, but the 95% CI (−8.5 to +1.9%) indicates this estimate is compatible with both meaningful benefit and trivial harm (Table 9).

**Table 9:** ARD for SAEs in individual trials.

Trial	Kwatra et al. (9)	Ständer et al. (7)	Ständer et al. (6)	PRIME2	PRIME
Outcome	−3.3%	+3.4%	−1.9%	+1.4%	−2.7%
95%CI	(−8.5%, 1.9%)	(−10.7%, 17.5%)	(−9.3%, 5.4%)	(−2.9%, 5.6%)	(−9.9%, 4.5%)

#### 3.5.3 AELTD

1. Sensitivity analysis using non-proportional hazards model (exponential model)

After adjustment using the non-proportional hazards model, the pooled risk ratio (RR) shifted from 0.89 to 1.21 (95% CI: 0.29–5.02), with substantial overlap in confidence intervals. This indicates that even under the assumption of exponentially increasing risk over time, the conclusions remained consistent, showing no statistically significant difference (*p* > 0.05).

2. Subgroup analysis (trials with 24-week duration only)

In the subgroup analysis restricted to 24-week trials, the RR suggested a marginal increase in risk (1.14), though the confidence interval (0.36–3.62) included the null value (RR = 1). The direction of this estimate contrasted with the primary pooled result (RR = 0.89). Potential explanations include:

Small-sample bias: Limited event counts (treatment group: 9 events vs. control: 4 events) led to unstable effect estimates.

Sources of heterogeneity: Shorter-duration trials (e.g., a 12-week trial with RR = 3.17) disproportionately influenced the pooled RR downward, though their own confidence intervals were extremely wide (e.g., 0.67–15.02), reflecting high uncertainty.

3. ARD analysis

In the Sonja trial for namilumab, a marginally higher rate of adverse events leading to treatment discontinuation (AELTD) was observed in the experimental group compared to the placebo group [absolute risk difference (ARD) = 2.9%]; however, the confidence interval (CI) included 0, indicating no statistically significant difference (Table 10).

**Table 10:** ARD for AELTD in individual trials.

Trial	Kwatra et al. (9)	Ständer et al. (7)	Ständer et al. (6)	PRIME2	PRIME
Outcome	−3.3%	+2.9%	+0.6%	−1.1%	−2.6%
95%CI	(−8.5%, 1.9%)	(−2.8%, 8.6%)	(−4.5%, 5.7%)	(−5.3%, 3.0%)	(−7.7%, 2.5%)

#### 3.5.4 Conjunctivitis

##### 3.5.4.1 Sensitivity analyses

1. Sensitivity analysis using non-proportional hazards model (exponential model)

The exponential model yielded a pooled RR of 0.75 (95% CI: 0.18–3.09). This result showed only a modest 8% difference compared to the linear model estimate (RR = 0.82), with overlapping confidence intervals and consistent directionality, collectively supporting the robustness of the linearity assumption.

2. Subgroup analysis (trials with 24-week duration only)

When restricted to 24-week trials, the RR was 0.17, suggesting a potential reduction in risk with Nemolizumab. However, the extremely wide confidence interval (0.01–4.14) and zero events in the treatment group rendered the results highly uncertain. The contrasting direction compared to the primary analysis (pooled RR = 0.75) may stem from: Zero-event issue: The absence of events in the treatment group (0 events) caused extreme instability in RR estimation. Sources of heterogeneity: Shorter-duration trials (e.g., 12- and 16-week trials with RR > 1 indicating a trend toward increased risk) diverged directionally from the 24-week trial result, though none reached statistical significance.

3. ARD analysis

In the PRIME2 trial, dupilumab showed an absolute risk difference of +3.9% for conjunctivitis (95% CI: −0.4 to +8.2%) with only 6 events observed. Given the wide confidence interval spanning both harm and protective effects (RR = 2.01, 0.29–13.77), this numerical imbalance does not support causal inference but may inform monitoring protocols in populations with preexisting ocular comorbidities (Table 11).

**Table 11:** ARD for conjunctivitis in individual trials.

Trial	Kwatra et al. (9)	Ständer et al. (7)	Ständer et al. (6)	PRIME2	PRIME
Outcome	+0.5%	+3.26%	−1.05%	+3.9%	0%
95%CI	(−0.5%, 1.6%)	(−8.9%, 15.4%)	(−3.1%, 1.0%)	(−0.4%, 8.2%)	(−5.2%, 5.2%)

#### 3.5.5 Alternative model verification

Through Bayesian and Beta-binomial model sensitivity analyses, we observed that: The continuity correction may underestimate the true variance (e.g., for the edema risk ratio: 1.03 in the original model vs. 1.01 in the Bayesian method); RR: 1.01 (0.01–102.4). The beta-binomial model fails to converge in double-zero event studies (e.g., the edema data from the Nemolizumab group), highlighting methodological limitations in analyzing ultra-sparse data. Bayesian credible intervals spanning multiple orders of magnitude (e.g., edema 95% CrI: 0.01–102.4) demonstrate that statistical “non-significance” does not equate to clinical equivalence. Although sensitivity analyses demonstrate the robustness of conclusions for primary outcomes (e.g., AELTD risk), the extremely wide credible intervals for zero-event outcomes like edema underscore the need for clinical vigilance against extreme risks in single-study samples. Real-world data are essential to supplement these findings, particularly for long-term medication use. For outcomes dominated by zero events (e.g., edema), Bayesian models yield exceptionally broad credible intervals (RR = 1.01 [0.01–102.4]), reflecting inherent uncertainty in sparse event modeling, albeit directionally consistent with the primary results (Table 12).

**Table 12:** Alternative model verification analysis.

Model	Original model	Bayesian models	Beta-binomial
Dupilumab AELTD	RR:0.42 (0.08–2.12)	RR:0.47 (0.05–4.32)	RR:0.48 (0.04–3.90)
Dupilumab edema	RR:1.03 (0.15–7.32)	RR:1.01 (0.01–102.4)	Model does not converge

#### 3.5.6 Baseline heterogeneity adjustment and impact of itch assessment tools

Meta-regression identified baseline itch severity (*β* = 0.062 per 1-point increase, *p* = 0.027) and itch assessment tools (WI-NRS vs. PP-NRS: β = 0.127, *p* = 0.032) as independent modifiers of adverse event (AE) risk.

*Stratified analyses*: The WI-NRS group showed a nominally higher AE risk point estimate (RR = 1.17 vs. 1.13 with PP-NRS), though the between-group difference was not statistically significant (*p* = 0.28).

*Sex effect*: A higher proportion of female participants was weakly associated with reduced AE risk (β = −0.005 per 1% increase, *p* = 0.012).

After adjusting for baseline heterogeneity, indirect comparisons demonstrated a diminished risk difference between the two agents (adjusted RR = 1.05, 95% CI: 0.82–1.34).

## 4 Discussion

### 4.1 Key findings and uncertainties

#### 4.1.1 Limitations in frequency and severity of adverse events

This exploratory analysis based on the Bucher indirect comparison framework assessed safety profiles between nemolizumab and dupilumab. The relative risk (RR) of 1.11 (95% CI: 0.85–1.47) for overall adverse events (AEs), indicating no statistically significant difference between the two biologics. However, numerical differences were noted in absolute risk metrics. All observed numerical differences should be interpreted as hypothesis-generating signals rather than confirmatory evidence, given their lack of statistical significance and overlapping confidence intervals. Notably, the wide CI spanning the null value in the indirect RR comparison (0.85–1.47) underscores substantial uncertainty in comparative AE risks. Furthermore, the absence of severity-stratified data (e.g., CTCAE grading) precludes clinical interpretation of frequency-based outcomes. For instance, nemolizumab’s significant ARD may be driven by transient mild events (e.g., injection-site reactions), while dupilumab’s CI spanning benefit and harm could reflect heterogeneous risk profiles combining low-grade conjunctivitis with potential rare serious AEs. These exploratory findings do not establish clinically meaningful risk differentials. Treatment decisions should weigh individual patient factors against the unquantifiable uncertainty inherent in indirect comparisons.

#### 4.1.2 Indirect comparisons of SAEs and AELTD

##### 4.1.2.1 Analysis of serious adverse events

The indirect meta-analysis revealed no statistically significant differences in SAE risk between nemolizumab (RR = 0.77, 95% CI: 0.43–1.39) or dupilumab (RR = 0.83, 95% CI: 0.25–2.76) versus placebo. In their respective trials, the absolute risk differences were not statistically significant indirect comparisons of ARDs across trials are methodologically inappropriate due to heterogeneity in placebo-group event rates and trial designs. The adjusted indirect RR between treatments (RR = 0.95, 95% CI: 0.34–2.68) further confirmed the absence of statistically significant differences. Sensitivity analyses using non-proportional hazard models and subgroup analyses restricted to 24-week trials consistently demonstrated non-significant outcomes (all *p* ≥ 0.05), reinforcing that current evidence does not support comparative conclusions.

##### 4.1.2.2 Analysis of adverse events leading to treatment discontinuation

Pooled analyses demonstrated non-significant risk differences for AELTD between nemolizumab (RR = 0.80, 95% CI: 0.32–1.96) and dupilumab (RR = 0.42, 95% CI: 0.08–2.12) versus placebo. Importantly, the outlier signal in the 2020 nemolizumab trial (RR = 3.17, 95% CI: 0.13–75.28) reflects extreme sampling variability due to low event counts (n = 1 event in active arm), highlighting the inherent uncertainty in interpreting rare AELTD events across trials.

Current evidence does not demonstrate differential safety profiles between these biologics regarding SAEs or treatment discontinuation risks. Prescribers should weigh these null findings against established efficacy benefits when making therapeutic decisions.

### 4.2 Mechanism-specific risk hypotheses

#### 4.2.1 Edema risk

The observed numerical difference in edema events with nemolizumab [absolute risk difference (ARD) = 0.062–0.190/100 person-weeks, +1.17%], corresponding to a number needed to harm (NNH) of 85, requires cautious interpretation within rigorous methodological constraints despite time-corrected sensitivity analyses. While the pathophysiological rationale suggests IL-31 receptor antagonists may theoretically induce fluid retention through neurovascular modulation pathways (16–19). The current evidence remains exploratory due to multiple limitations. Statistically, the substantial overlap in risk ratio confidence intervals [RR = 1.89, 95% CI 0.52–5.18; sensitivity analysis RR = 2.54 (0.30–21.43)] indicates susceptibility to type I/II errors. Methodological concerns include non-standardized fluid retention assessment protocols that risk detection bias amplification, challenges in differentiating random event clustering from true drug effects given low placebo-group event rates (0–2 cases/group) and treatment duration heterogeneity (12–24 weeks), and potential model misspecification arising from the linear time-effect assumption conflicting with nonlinear drug accumulation patterns. Clinically, even if accepting signal validity, even if a true risk exists, the estimated population-level risk elevation is minimal [ARD<0.2/100 person-weeks, number needed to treat (NNT) = 1,610–526], likely below clinical management significance.

This mechanistic-methodological paradox underscores the necessity for prospective validation through pre-specified fluid monitoring protocols, standardized treatment durations, and expanded sample sizes to resolve current evidentiary uncertainties.

#### 4.2.2 Conjunctivitis risk

Current evidence indicates that for nemolizumab, no conjunctivitis events were observed in the 24-week trial (0/187 vs. 1/95 in placebo), though 12-week data revealed a non-significant numerical difference with an upward trend in relative risk (RR = 1.59, 95%CI:0.28–8.93), potentially related to early exposure dynamics. The absolute risk difference (ARD) exhibited an asymmetric distribution. For dupilumab, exploratory analyses showed numerically elevated but statistically non-significant conjunctivitis risk at 24 weeks (ARD = +3.89%, 95%CI:-0.4% to +8.2%; RR = 2.01, 95%CI:0.29–13.77). The ultra-wide confidence intervals spanning both potential harm (upper bound: RR = 13.77) and protection (lower bound: RR = 0.29), coupled with the absence of statistical significance, strictly preclude causal inferences. While the observed numerical imbalance theoretically aligns with IL-4/IL-13 pathway inhibition effects on ocular mucosal immunity (20), this hypothesis-generating signal must be interpreted as an exploratory observation requiring validation in prospective trials with protocol-driven ophthalmic monitoring. These analyses collectively emphasize that numerical differences even those mechanistically plausible do not constitute confirmatory evidence of risk and should serve solely to inform future hypothesis-testing studies.

### 4.3 Absolute risk differences: clinical interpretation and precision limitations

This study quantified the safety profile of nemolizumab and dupilumab relative to placebo using absolute risk difference (ARD), though precision was limited by the following factors:

#### 4.3.1 Clinical interpretation of confidence intervals

Nemolizumab showed heterogeneous AE risk across trials. Specifically, the most notable absolute difference was observed in Ständer 2025 trial (*n* = 282, ARD+8.9% with placebo AE rate 65.3%), while the paradoxical result in Ständer 2020 trial (*n* = 70, ARD-5.6% with placebo rate 66.7%) may reflect limited sample size. Although the upper CI limit suggests potentially clinically relevant differences in high-risk scenarios, it’s crucial to emphasize this estimate should not be extrapolated across trials.

#### 4.3.2 Low event rates and statistical power limitations

The rarity of mechanism-related events (e.g., edema, conjunctivitis) led to imprecise ARD estimates. For example, nemolizumab’s edema ARD was +1.17% (95% CI: −1.24 to 3.58%), with a number needed to treat (NNH) of 85 to result in one additional edema case. The clinical relevance of such findings may depend on the severity of the outcome.

#### 4.3.3 Baseline heterogeneity and clinical implications of pruritus assessment tools

This study examined the independent effects of baseline pruritus scores and assessment tools on AE risks:

*Pruritus intensity and risk association*: Higher baseline pruritus scores (regardless of WI-NRS or PP-NRS) were associated with increased AE risks (+6.2% per unit), which may correlate with more severe skin barrier disruption and neuroimmune activation in patients with intense pruritus.

*Differences in assessment tools*: The AE risk point estimate was higher in the WI-NRS group (dupilumab trials) compared to the PP-NRS group (nemolizumab trials). This discrepancy might arise because WI-NRS captures “worst-itch moments,” potentially reflecting short-term inflammatory fluctuations, whereas PP-NRS measures “average intensity,” possibly aligning more closely with chronic pathological burden.

### 4.4 Study limitations

#### 4.4.1 Risk of Bias in indirect comparisons

Baseline heterogeneity across trials (e.g., variability in prurigo duration) may influence outcomes in indirect comparative analyses.

#### 4.4.2 Lack of AE severity grading

A critical limitation of this study is the absence of CTCAE (Common Terminology Criteria for Adverse Events) severity grading across all included trials. The inability to stratify adverse events by severity (e.g., mild, moderate, severe) significantly constrains the clinical interpretation of safety profiles. For instance: Frequency-Severity Discrepancy: Equivalent overall AE rates between dupilumab and nemolizumab may mask divergent clinical impacts. Dupilumab’s observed conjunctivitis trend (RR = 2.01) might primarily involve mild, self-limiting cases (e.g., Grade 1: transient irritation), whereas nemolizumab’s edema signals (RR = 1.64) could represent Grade 2–3 events requiring therapeutic intervention. Such distinctions are critical for risk–benefit assessments in vulnerable populations (e.g., patients with preexisting ocular surface disease or cardiorenal comorbidities). Misinterpretation of Safety Signals: Severe but infrequent AEs (e.g., dupilumab-associated systemic infections or nemolizumab-induced angioedema) may be underestimated in frequency-based analyses, while high-frequency mild AEs (e.g., injection-site reactions) could overstate perceived risks. This creates a false equivalence in safety comparisons. Impact on Clinical Decision-Making: Without severity data, clinicians cannot prioritize interventions based on AE criticality. For example, a higher incidence of mild conjunctivitis may be clinically acceptable if balanced against lower risks of severe edema, but this trade-off remains unquantifiable in the current evidence.

*Proposed solutions for future trials*: Mandate CTCAE-Aligned Grading: All mechanism-specific AEs (e.g., conjunctivitis, edema) should be reported with CTCAE severity grades (Grades 1–5) and duration-adjusted event rates (e.g., events per 100 person-weeks by grade). Develop Composite Metrics: Integrate frequency and severity into weighted scores [e.g., AE severity index = *Σ* (Grade × Events)/Person-Time] to better reflect cumulative burden. Report Clinical action ability: Explicitly categorize AEs by their management requirements (e.g., “self-resolving,” “requiring topical therapy,” “leading to hospitalization”).

#### 4.4.3 Sensitivity of zero-event handling

##### 4.4.3.1 Potential bias from zero-event corrections

Although continuity corrections (e.g., adding 0.5 to all cells of 2 × 2 tables) were applied to handle zero-event studies in RR calculations, this approach may introduce the following biases:

*Underestimation of rare-event risks*: In extreme scenarios where placebo arms report zero events (e.g., SAEs or AELTDs), continuity corrections may underestimate true risk differences. For instance, if a trial reports 1 event in the treatment arm versus 0 in placebo, the corrected RR would be artificially reduced (from infinity to 3.0), leading to effect dilution.

*Sensitivity to event rates*: When event rates are <5%, corrected RR estimates become highly sensitive to minor numerical adjustments (e.g., ±0.5 event shifts may cause >20% RR fluctuations).

*Directional bias risk*: If zero-event distributions are imbalanced across trials (e.g., fewer AELTD events in dupilumab arms), pooled RRs may be skewed toward the null (e.g., AELTD RR = 0.42–0.89 in this study).

Although the main results were validated through Bayesian and Beta-binomial models, both models—particularly the Beta-binomial model—failed to converge in double-zero event studies, highlighting the methodological limitations of analyzing ultra-sparse data. Additionally, the Bayesian credible intervals spanned orders of magnitude (e.g., edema: 95% CrI: 0.01–102.4), indicating that a lack of statistical “significance” does not equate to clinical equivalence.

#### 4.4.4 Extrapolation error in exposure duration

Extrapolating short-term nemolizumab data to 24-week exposure assumes non-proportional hazards (i.e., constant hazard ratios over time), which may overestimate differences if treatment effects vary with duration.

#### 4.4.5 Potential influence of pruritus assessment tools

While meta-regression adjusted for pruritus assessment tools (WI-NRS vs. PP-NRS), residual confounding may persist due to unmeasured dimensional heterogeneity (e.g., “worst-itch moments” vs. “average intensity”). Additionally, shorter disease duration in WI-NRS trials (mean 5.4–5.7 years vs. 7.6–8.8 years in nemolizumab trials) could confound the relationship between disease chronicity and AE risks.

### 4.5 Clinical implications

Given wide confidence intervals (e.g., dupilumab’s AE ARD spanning the null value), clinical decisions should be balanced against the biological plausibility of mechanism-related risks (e.g., IL-4Rα inhibition and conjunctivitis) and patient-specific factors (e.g., cardiorenal status). Enhanced monitoring protocols (e.g., bi-weekly edema/conjunctivitis assessments during early treatment) are advisable for high-risk populations to detect safety signals potentially exceeding current ARD estimates. In the absence of severity data, we recommend: Risk-Adapted Monitoring: For patients with predisposing factors (e.g., glaucoma, congestive heart failure), proactively screen for mechanism-specific AEs (e.g., monthly ocular exams for dupilumab, fluid status assessments for nemolizumab) regardless of overall AE frequency. Shared Decision-Making: Counsel patients on the uncertainty of severity-specific risks, emphasizing that “common” AEs may not correlate with severity.

### 4.6 Clinically meaningful ARD thresholds in PN treatment: expert perspective

The determination of a clinically meaningful threshold for Absolute Risk Difference (ARD) in prurigo nodularis (PN) requires a multifactorial assessment that integrates therapeutic context, disease burden, and patient priorities. While no universal numerical threshold exists, the FDA guidance (21) emphasizes that regulatory decisions weigh benefits and risks within the framework of.

#### 4.6.1 Disease severity

PN is a chronic, debilitating condition characterized by intractable itch, sleep disruption, and reduced quality of life. Patients often have severe disease refractory to conventional therapies. In such high-need populations, higher ARD thresholds for adverse events (AEs) may be acceptable if accompanied by robust efficacy (e.g., sustained itch reduction or lesion clearance).

#### 4.6.2 Nature and impact of benefits

Trials of dupilumab and nemolizumab demonstrate clinically significant efficacy: Dupilumab: WI-NRS improvement ≥4-point (60% dupilumabvs 18.4% placebo) (8). Nemolizumab: PP-NRS ≥ 4-point reduction (56.3% nemolizumab vs20.9% placebo) (9). FDA considers endpoints reflecting direct patient benefit (e.g., itch reduction, functional improvement) as critical to offset AE risks.

#### 4.6.3 Risk profile contextualization

**O**verall AEs: The ARD for nemolizumab ranged from +1.0% to +8.5% across trials. In PN, this risk may be acceptable given the high unmet need.

#### 4.6.4 Mechanism-specific risks

Conjunctivitis (dupilumab: ARD 0–3.89%) typically involves mild, reversible cases, whereas edema (nemolizumab: ARD +1.17%) may require monitoring in cardiorenal comorbidities. These risks are generally manageable with routine care.

### 4.7 Future research directions

Standardized AE Severity Grading: Head-to-head trials should pre-specify CTCAE criteria to differentiate severity levels and clinically meaningful endpoints (e.g., treatment discontinuation, hospitalization, or irreversible harm). Consensus-Driven SAE Assessments: Future trials require standardized protocols for serious AE (SAE) evaluations (e.g., validated edema grading systems). Extended Follow-Up for Delayed Events: Prolonged observation periods are needed to identify delayed-onset risks (e.g., dupilumab-associated infections). This study reveals that the selection of pruritus assessment tools (WI-NRS versus PP-NRS) may indirectly influence safety outcome analyses by reflecting distinct disease activity patterns. The WI-NRS-captured acute pruritus peaks potentially correlate with transient Th2-mediated inflammatory surges, corresponding to an initial increased risk of conjunctivitis (RR = 2.01) during early treatment phases. Conversely, PP-NRS-assessed chronic pruritus burden appears more dependent on IL-31-mediated neuroimmune interactions, potentially extending the monitoring window for delayed adverse events (AEs) such as edema (ARD = +1.17%). Although meta-regression analysis indicated an independent association between assessment tool selection and AE risk (*β* = 0.127, *p* = 0.032), residual confounding factors require cautious interpretation - notably, the inherently higher atopic comorbidity burden in WI-NRS trials might intrinsically predispose to ocular AEs. Future investigations should integrate multidimensional pruritus profiling with biomarker stratification (e.g., serum IL-31 levels) to differentiate methodological artifacts (“noise”) from genuine pathophysiological signals in safety outcome assessments. Future directions propose pre-specified composite endpoints in head-to-head trials encompassing: (1) safety composites (e.g., discontinuation rates, irreversible injury incidence, cumulative ≥Grade 3 AE risks); (2) patient-reported outcomes (PROs) integrating simultaneous WI-NRS/PP-NRS measurements with temporal itch-alleviation patterns to elucidate dynamic AE correlations; and (3) biomarker-guided risk stratification combining neuroimmune markers (e.g., IL-31) with traditional Th2 cytokines to enhance AE predictability. Cross-validation of multidimensional data (clinical endpoints, PROs, biomarkers) will improve discrimination between methodological artifacts and pathophysiology-driven safety signals.

## 5 Conclusion

Baseline pruritus severity scores and their assessment tools (WI-NRS and PP-NRS) may serve as potential predictors for adverse event (AE) risk. Indirect treatment comparisons revealed no significant differences in overall safety profiles between the agents, though the dupilumab group showed numerically higher risk estimates (clinical relevance undetermined), suggesting future research should validate itch-based monitoring strategies. Subsequent studies must standardize endpoint definitions and adjust for disease course heterogeneity to minimize confounding effects. Adjusted indirect comparisons demonstrated comparable overall AE risks between dupilumab and nemolizumab for prurigo nodularis treatment (indirect RR = 1.11, 95%CI 0.85–1.47). Mechanistic exploratory endpoint analyses warrant cautious interpretation: Dupilumab showed a non-significant trend toward higher conjunctivitis risk [RR = 2.01(0.29–13.77)], potentially aligning with IL-4/IL-13 pathway inhibition theory, though current evidence remains insufficient to establish causality or exclude random variation. Nemolizumab was associated with numerically higher standardized incidence rates for edema (0.112–0.245 vs. 0.050–0.055 per 100 person-weeks), yet overlapping confidence intervals suggest differences may reflect random variability. Crucially, the absence of statistically significant AE rate differences does not establish clinical safety equivalence. Fundamental limitations in interpreting AE clinical relevance exist due to missing CTCAE grading data. Future head-to-head trials must incorporate severity-stratified reporting to differentiate “mildly bothersome” (Grade 1–2) from “clinically significant” (≥Grade 3) events. Serious adverse event (SAE) rates showed no statistical divergence between groups. Study limitations include: 1. Trial design heterogeneity (treatment duration, endpoint definitions), 2. Low incidence rates for critical events, and 3. Lack of AE severity grading precluding assessment of clinically meaningful event burdens. Phenotype-specific risk discussions following exposure time and regional variation adjustments require explicit hypothesis-generation framing: Cardiorenal Comorbidities: Despite theoretical considerations, dupilumab’s presumed safety advantages in PN patients with cardiorenal dysfunction require prospective subgroup validation, particularly regarding IL-4/IL-13 pathway inhibition’s potential cardiovascular effects. IL-31 Antagonism & Edema Monitoring: Nemolizumab’s IL-31 pathway blockade may benefit atopic phenotypes but necessitates intensified fluid retention monitoring during treatment initiation, especially in patients with chronic kidney disease or heart failure. The observed marginal elevation in edema rates (absolute risk difference +1.17%) shows exploratory alignment with preclinical hypotheses of IL-31-mediated vascular effects, though biological and clinical relevance requires pharmacodynamic validation.

## Data Availability

The original contributions presented in the study are included in the article/supplementary material, further inquiries can be directed to the corresponding author/s.
